# Secure and Trusted Crowdsensing for Outdoor Air Quality Monitoring: State of the Art and Perspectives

**DOI:** 10.3390/s25123573

**Published:** 2025-06-06

**Authors:** Claudio Marche, Emmanuele Massidda, Alessandro Sanna, Gianmarco Angius, Michele Nitti, Davide Maiorca, Stefano Lai

**Affiliations:** 1 Department of Electrical and Electronic Engineering (DIEE), University of Cagliari, 09123 Cagliari, Italy; emmanuele.massidda@unica.it (E.M.); alessandro.sanna96@unica.it (A.S.); gianmarco.angius@unica.it (G.A.); michele.nitti@unica.it (M.N.); davide.maiorca@unica.it (D.M.); stefano.lai@unica.it (S.L.); 2 National Inter-University Consortium for Telecommunications (CNIT), University of Cagliari, 09123 Cagliari, Italy

**Keywords:** outdoor air quality monitoring, mobile crowdsensing, secure, trusted, health, environment

## Abstract

Air pollution is a major problem in the modern world; although it particularly impacts developing countries, which are experiencing fast and often uncontrolled industrialization, its effects constitute a global burden on the environment and health. At the same time, the costs of effective air quality monitoring programs are prohibitive for emerging economies, thus making any correction difficult to assess. Emerging technologies, such as distributed networks of sensors organized in the Internet of Things, are under the lens of scientific and industrial communities as a valuable, low-cost alternative to standard techniques. In this paper, we report a review of current approaches to distributed air quality monitoring. Specifically, we *(1)* emphasize the role of crowdsensing in leveraging sensor-enabled mobile devices for large-scale environmental data collection and *(2)* discuss criticalities, open challenges, and future perspectives in enforcing data security when such approaches are deployed in real application scenarios.

## 1. Introduction

In the last 200 years, humanity has experienced the most dramatic technological development of its history, mostly guided by a succession of industrial revolutions until the present day. It is undeniable that the incredible advantages enabled by the industrial era are counterbalanced by several drawbacks [1]; therefore, in parallel with the definition of strategies towards sustainable development, the management of the current situation plays a fundamental role in balancing social and public health aspects [2]. Aside from great environmental upheavals, endemic consequences of human activities must be tracked. Among other methods [3], monitoring air quality is essential to evaluate the impact of pollution on several aspects, such as climate change, the environment, and human health. As a result of transportation, energy production, industrial and agricultural activities, indoor air pollution, and household appliances such as HVAC (Heating, Ventilation, and Air Conditioning) systems, the concentration of pollutants due to these usually overcomes the one related to natural causes, including wildfires, volcanic activity, and dust storms. In specific areas and at specific times of the year, the concentration of air pollutants becomes easily dangerous for health, especially for vulnerable populations such as children, the elderly, and people with respiratory or cardiovascular conditions. Moreover, air pollutants damage the environment, crops, and vegetation and seriously harm wildlife. Last but not least, uncontrolled concentrations of some specific pollutants, such as carbon monoxide and dioxide, are among the leading players in climate change.

Regarding the state of the art, the main instrument to track air quality is the installation of monitoring stations in specific areas in which high levels of pollution are somehow expected, such as crowded metropolitan areas or regions near industrial facilities. Since several high-resolution instruments can be used in these stations, qualitatively accurate data can be retrieved, but with a low spatial resolution and significant installation and maintenance costs. This represents a barrier for the thorough monitoring of air quality in emerging economies that may find affording these costs difficult, even if they are by far those which would benefit most from it (with their industries strongly reliant of fossil fuels). For these reasons, communities worldwide are increasingly pushing for developing and implementing distributed air quality monitoring protocols. Air pollution monitoring using portable or distributed networks of sensors has been an active research area in recent years, involving different expertises in the field of information and communication technology (ICT) [4]. In particular, the miniaturization of sensors and reductions in power consumption represent fundamental features for any portable system. The current development of electronic technologies has enabled several possibilities in this sense, and indeed, miniaturized sensors for common air pollutants are available on the market at affordable costs for the development of customized systems. The trade-off of cost-effectiveness and portability is represented by averaging measurement outputs coming from several sensors, but this poses specific challenges in terms of hardware resources, networking, and data security. Moreover, in order to ensure appropriate spatial resolution and data reliability, a significant number of sensors should perform acquisitions in a given area and in a limited amount of time, thus requiring novel paradigms beyond the usual, solicited measurement by technicians and operators.

In this paper, a review of the more recent literature on distributed sensor networks for outdoor air quality monitoring is reported. A multi-disciplinary approach will be followed to shine a light to every fundamental aspect of this particular application field. First, main air pollutants will be discussed, with a particular emphasis given to the health and socioeconomical burdens of air pollution. Then, the main technological aspects in the development of distributed networks of sensors will be reported, with particular attention devoted to crowdsensing approaches and data security aspects. Open challenges and main opportunities will be analyzed, in order to define possible enabling approaches for the actual exploitation of these approaches in real application scenarios and at a market level.

## 2. Distributed Air Quality Monitoring Systems: Health and Socioeconomical Perspectives

Outdoor air quality has a profound influence on human health and social well-being, making it a critical factor in environmental health assessments. In 2019, the World Health Organization (WHO) estimated that 4.2 million premature deaths per year were related to the air pollution, with a prevalence of deaths (89%) in low- and middle-income countries. In the same year, globally, 99% of the population was estimated to live in areas where the maximum concentration of air pollutants exceeded the air quality guidelines established by the same WHO [5]. The better management of air quality, as allowed by the more specific evaluation of critical areas with novel approaches to air pollutant monitoring, can significantly impact the socioeconomical fabric. Conti et al. estimated that in Europe alone, complying with WHO guidelines would save EUR 31 billion per year (EUR 19 million only related to hospitalizations) [6].

To properly analyze the positive impact of distributed air quality monitoring systems, the main air pollutants affecting human health and their origins must be introduced. Air pollution is a complex scenario of strongly interconnected components and reactions, with variable sources according to geographic areas and context. Considering exposure to general population, HVAC systems and road transport are the main sources of the primary constituents of air pollution, such as particulate matter (PM), nitrogen dioxide (NO_2_), ozone (O_3_), carbon monoxide (CO), benzene and other Volatile Organic Compounds (VOCs). Moreover, specific environmental conditions, such as heath and sunlight exposure, may contribute to the secondary generation of pollutants; for instance, O_3_ can originate from photochemical reactions between VOCs and nitrogen oxides. Interestingly enough, these reactions can be also generated by the interaction between anthropogenic pollutants and biogenic VOCs, i.e., VOCs originating from vegetation [7]. Similarly, NO_2_ interaction with BVOCs can significantly contribute to the generation of PM. Other pollutants, such as sulfur dioxide (SO_2_), mostly originate from industrial activity. The health burdens of air pollution ranges in short-term and long-term scenarios. Air quality significantly impacts pre-existent pathologies, such as asthma, respiratory infections, and cardiovascular diseases, and contributes to reduced lung functions for specific population categories, such as children and the elderly [8,9,10]. In the last twenty years, several studies have analyzed the impact of air pollutants on the insurgence of specific diseases, in several contexts and in various conditions. In the more recent reports, the combined effects of major pollutants have been analyzed: high levels of pollution strongly impact small airway diseases [11] and mortality by cardiovascular and respiratory diseases and lung cancers [12]. Nonetheless, each pollutant has its own specific impact on health, related to its physiochemical characteristics. It is normally accepted that, among air pollutants, PM is the most dangerous for human health, due to its capability of deep lung penetration and access to the bloodstream, inducing cardiovascular (ischaemic heart disease), cerebrovascular (stroke), and chronic respiratory diseases (e.g., chronic obstructive pulmonary disease, COPD). Both long-term [13,14] and short-term [15] exposure to PM is associated with morbidity and mortality from cardiovascular and respiratory diseases. Long-term exposure has been further linked to adverse perinatal outcomes [16] and lung cancer [17]. Similarly, the effect of VOC exposure has been reported as significantly enhancing all-cause and specific-cause mortality rates [18], with a clear impact (among various ones) on cancerogenesis [19], endothelial damage and reduced healing [20], and ischemic heart diseases [21]. The most up-to-date data diffused by the WHO indicate that air pollution as a whole accounts for 43% of deaths and diseases from chronic obstructive pulmonary diseases, 25% of deaths and diseases from ischaemic heart disease, 24% of deaths from stroke, 17% of deaths and diseases from acute lower respiratory infection, and 29% of deaths and disease from lung cancer. The role of air pollution in the morbidity of certain diseases has a clear impact on several aspects in the economical and health–social fabric. The burden of air pollution in this context is related (but not limited) to (i) reduced production, due to work leave for disease; (ii) anticipated retirement due to severe illness, with an impact on pension insurance; (iii) augmented access and hospital stays for air pollution-related diseases; (iv) premature death. It is noteworthy that aspects directly related to air quality have been listed here, but these must be counted together with other socioeconomical impacts, such as those related to the climate change. An overall scenario of air pollution health and socioeconomical impact is hard define: Lanzi et al. estimated that, by 2060, the overall healthcare cost will be about USD 142 billion, i.e., ten times larger than that in 2010 and approaching the 1% of global Gross Domestic Product (GDP) [22]. A recent study by Romanello et al. reports monetary costs of premature deaths related to air pollution in 2020 of USD 2.2 trillion, which was equivalent to 2.4% of the global GPD [23].

The European Union [24] and the World Health Organization [5] provide threshold values for the main outdoor air pollutants (Table 1). WHO limits are generally more restrictive than EU ones (with the sole exception of SO_2_), thus making a significant percentage of Europeans living significantly above the WHO indications [24]; in the case of PM_2.5_, the percentage approaches 96%, and for PM_10_, it approaches 83%. Similar results are found on O_3_ (94%) and NO_2_ (88%). The new zero-pollution action plan [25] aims to significantly adjust these results to the WHO previsions by 2030 and to reach the ambitious results of neutralizing significant impacts on health by 2050 [26], thus requiring significant effort in the spatial and time resolution of data acquisition [27].

The complex scenario of air pollution monitoring and management in the modern era requires the significant evolution of measurement systems. Indeed, traditional air quality monitoring networks relying on a limited number of fixed stations, fail in terms of spatial coverage and flexibility, although they still remain the gold standard for regulatory compliance purposes due to high data accuracy and reliability. On the contrary, distributed air quality monitoring systems, composed of low-cost and portable sensors, potentially offer several advantages, particularly relevant for understanding and mitigating the health, environmental, and societal impacts of air pollution, thus being a valuable solution complementary to standard monitoring systems.

One of the primary strengths of distributed systems is their capacity to generate high-resolution data both spatially and temporally. Fixed stations are generally sparsely distributed due to their high cost and maintenance requirements, which results in a lack of detailed information at the neighborhood or street level. By contrast, distributed systems can be deployed across a dense network, capturing localized pollution patterns and enabling the identification of hotspots that would otherwise go undetected. Additionally, many portable sensors offer high-frequency measurements, making it possible to track short-term pollution episodes—such as rush hour traffic peaks or industrial releases that might be averaged out in the data from conventional stations. Such a specific evaluation of air quality allows the better management of exposure conditions, thus representing a valuable instrument for monitoring the effectiveness of pollution mitigation strategies in a more comprehensive way. In the context of public health, distributed monitoring not only allows the evaluation of the short- and long-term exposure of general citizens, but it can even be extended towards more accurate and personalized exposure assessment. Wearable or mobile sensors can track an individual’s exposure across different environments and over time, offering a far more realistic picture than static measurements from fixed stations. This is particularly valuable for vulnerable populations, including children, the elderly, pregnant women, and individuals with chronic respiratory or cardiovascular diseases. More precise exposure data can improve the quality of epidemiological studies and support targeted public health interventions. Another important advantage lies in addressing inequalities in air pollution monitoring. Fixed stations are often installed in central or regulatory-prioritized areas, potentially neglecting low-income or marginalized neighborhoods that may experience higher pollution levels due to proximity to highways, industrial zones, or substandard housing conditions. Distributed systems can be deployed deliberately in these under-monitored areas, thus supporting environmental justice efforts and helping policymakers address disproportionate health burdens. Similarly, the relatively low cost and scalability of distributed monitoring technologies make them accessible for widespread implementation, particularly in regions with limited resources or where conventional monitoring infrastructure is lacking: these areas typically coincide with developing countries, which are indeed those more affected by air pollution issues.

## 3. Technologies and Approaches for Distributed Monitoring of Outdoor Air Quality

### 3.1. IoT and Crowdsensing for Air Quality Monitoring

In the present era of the Internet of Things (IoT), mobile crowdsensing (MCS) has attracted considerable attention from the scientific community [28]. MCS has gained significant recognition as an emerging paradigm for data sensing and collection. This approach leverages various sensors integrated into mobile devices like smartphones and wearables, which enable advancements across a wide range of fields, including healthcare, environmental monitoring, and traffic management [29].

**Figure 1:**
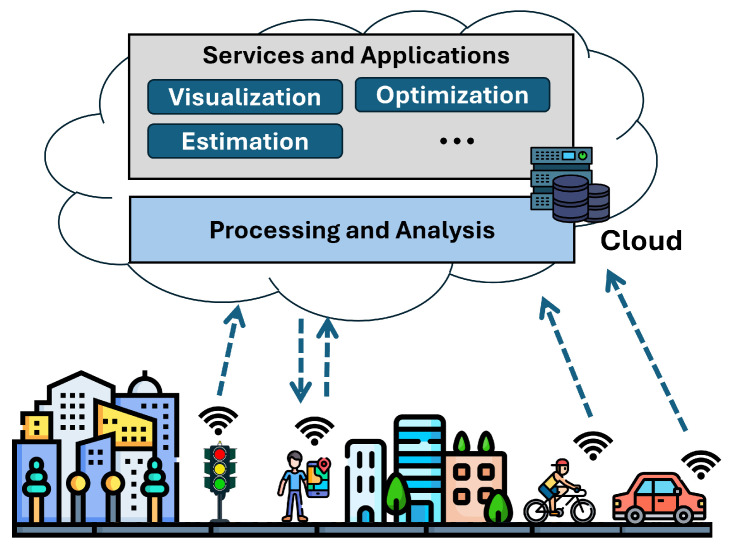
Scenario of a generic MCS system.

Figure 1 illustrates a generic scenario of an MCS system, where various devices communicate with the cloud by transmitting data collected from the real world. Specifically, different sensors attached to cars, buses, or bicycles send environmental measurement data, which is then analyzed and processed in the cloud. The cloud provides various services, such as monitoring and prediction, which users can access through personal devices like smartphones. These smartphones can also be utilized as part of the data collection campaign themselves.

In line with this, the following section offers a review of key studies that have contributed to the development of air quality monitoring within the MCS framework. In the following analysis, the aim is not to examine all the works in the state of the art but to focus exclusively on the most well-known and recent studies.

In recent years, MCS with vehicles for environmental monitoring has been explored in multiple studies. Among the works, in [30], the authors propose a vehicle sensor network for monitoring PM in urban areas. The system consists of equipping public buses and private cars with IoT sensors to collect air quality data in real time. These sensors measure geolocated PM concentrations and transmit the data to a centralized server. The data is then compared with reference data from governmental monitoring stations and displayed on a web platform. Similarly, other two works are proposed in [31,32]. The first work introduces an open data infrastructure using public buses. The data is transmitted through mobile networks, processed, and visualized in a web application. With the goal of reducing costs associated with air quality monitoring, the model applies statistical methods, such as standard deviation and coefficient of variation, to adjust for the accuracy limitations of the low-cost sensors, using reference measurements as a baseline. In the second work, the authors propose a framework using personal vehicles to monitor air quality and urban mobility in real time. The system includes onboard units with sensors that collect environmental and mobility data as vehicles move across the city. These data are transmitted via low-power wide-area network technologies to a server that performs processing using time series analysis and predictions of pollution and mobility patterns.

Another approach to crowdsensing involves portable devices. Two proposals are illustrated in [33,34]. In the first paper, the authors present an air quality monitoring architecture focusing on fixed and mobile sensors. The system supports monitoring in urban areas, collecting data on pollutants such as NO_2_ and PM. The collected data is processed to build predictive models for future air quality. In the second paper, the authors propose a system based on mobile crowdsensing using bike-share networks. The boxes with sensors are attached to shared bicycles, which gather data while moving through urban areas. The Bayesian compressive sensing model is used to fill in data gaps and reconstruct a complete map of urban air quality. Furthermore, another approach focused on portable devices is depicted in [35], in which the authors introduce an open-source platform that allows citizens to deploy IoT sensors for measuring particulate matter in urban environments. The system includes a data analysis platform and a method for remotely updating sensor firmware. The aggregated data is analyzed and visualized in a web view.

In addition to air quality, crowdsensing has also been applied to monitor temperature and noise pollution. Among these works, two are illustrated in [36,37]. The first paper presents a platform that allows citizens to use their smartphones to collect noise-level data within the city. The mobile app gathers data, pre-processes it, and sends it to a cloud-based infrastructure for storage and real-time analysis. Meanwhile in the second paper, the authors explore the use of smartphones to predict indoor ambient temperatures. Since smartphones lack external temperature sensors, the authors developed a machine learning model that leverages battery temperature data to estimate the ambient air temperature.

A more advanced form of data integration is explored in [38]. Specifically, the authors developed an AI-based sensor network aimed at assisting citizens with respiratory issues. The system collects data from a distributed network of IoT sensors that measure pollutants such as CO_2_, NO_2_, and PM. The data is processed using AI models, such as LSTM (Long Short-Term Memory) and CNNs (Convolutional Neural Networks), to predict future air pollution levels and identify potential respiratory hazards.

Finally, another important work is depicted in [39], where the authors propose a system designed to estimate missing environmental information in smart cities. The system integrates data from heterogeneous sensors distributed across an urban environment to monitor variables such as temperature, humidity, and CO_2_. The model estimates missing data by integrating historical values and data from external sources.

Beyond the architecture, another important aspect concerns the description, encoding, and processing of collected data. In almost all the reviewed works, data collected from mobile or fixed sensors is structured using standardized formats such as JSON, enabling the encapsulation of values with metadata (e.g., measurement unit, sensor type, timestamp, and geolocation). However, while this basic structuring is adopted to ensure data transmission, more advanced paradigms are still scarcely addressed. Only a few platforms begin to incorporate different forms of data annotation to increase interoperability. For instance, the system proposed in [33] adopts a JSON format with extended metadata and discusses the importance of coherence in long-term deployments. Moreover, most contributions include pre-processing stages like filtering or calibration and, in some cases, basic statistical correction but lack an explicit description of the full pipeline or sensors’ uncertainty.

To sum up, Table 2 summarizes the representative contributions described above and the classification of the analyzed works. The approaches are classified based on the types of objects used for sensing, the types of data collected, the sensing devices employed, how the collected data are utilized, and finally, the level of user involvement. Specifically, user involvement is categorized as participatory when users play an active role in the data collection, while opportunistic refers to passive contributions, where users indirectly or automatically provide data without explicit participation.

In general, all the analyzed approaches are innovative in leveraging MCS to reduce the high costs associated with precise air quality monitoring systems, which require significant investments in device installation and maintenance. By employing non-specialized available devices like smartphones or shared bikes equipped with basic IoT devices, these studies demonstrate cost-effective solutions that make environmental monitoring more accessible. MCS platforms commonly employ low-cost gas and particulate matter sensors, such as optical dust sensors (e.g., PMS5003 sensor (Plantower, Beijing, China) and SDS011 sensor (Nova Fitness Co., Jinan, China)) and electrochemical gas sensors (e.g., Alphasense series), due to their affordability and ease of integration. However, several used sensors are known to suffer from several limitations that affect data quality [40]. These include sensitivity to nontarget gases, environmental dependency (e.g., temperature and humidity), and sensor drift over time. As a consequence, the numerical output of these sensors does not necessarily correspond to accurate pollutant concentrations unless adequate calibration procedures are implemented. Some of the reviewed works attempt to mitigate this issue by applying statistical corrections or by referencing measurements from governmental monitoring stations. Nonetheless, sensor calibration and data validation remain open challenges for these systems.

Furthermore, another important component is represented by visualization. As illustrated, in most of the reviewed approaches, data visualization is provided through web-based dashboards that display information over maps or statistical summaries of pollutants. These interfaces often aggregate data according to geographic zones, facilitating trend analysis and anomaly detection. The type of data displayed is related to the structure of the data collected, as introduced in Section 2. For instance, measurements of PM_2.5_ or NO_2_ are represented using heatmaps, while metadata such as sensor location or device ID are used to filter the visual output. In some systems, user-friendly summaries, such as traffic-light indicators or air quality indexes, are also illustrated. Despite their usefulness, visualizations are often not described in technical detail.

Finally, another important concern is represented by the trustworthiness of the data collected by volunteers. Most reviewed contributions rely on passive data collection mechanisms, where volunteers contribute data opportunistically without extensive control over individual behaviors. Consequently, very few works explicitly address the consistency of participant engagement or introduce a dedicated trust assessment. In general, data validation is implicitly ensured through aggregation, redundancy, and comparison with reference stations when available. Few systems, such as in [31,33], adopt statistical correction techniques to mitigate errors; however, methods for evaluating the reliability of the participants, such as trust scoring and reputation, are largely absent.

### 3.2. Security Issues of Distributed Monitoring Applications

**Figure 2:**
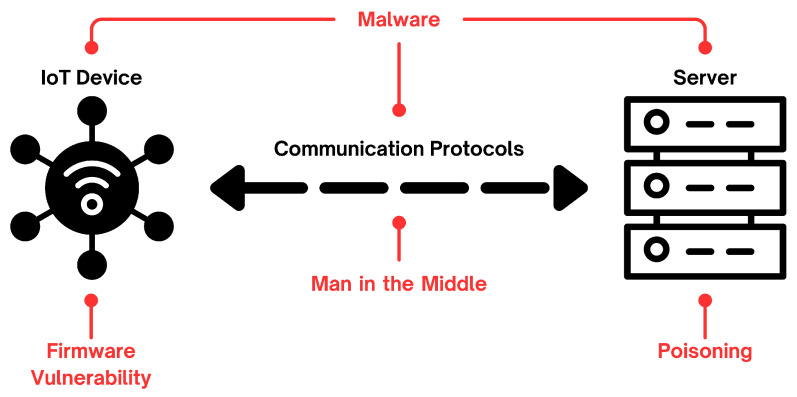
Graphical representation of the attack surface.

In the current landscape of MCS considered in Section 3.1, consideration for the security aspect has been scarce. Among the cited works, only three of them [34,35,38] discuss the topic. This lack of attention is concerning, as ensuring the integrity and authenticity of crowdsensed data is essential. Without proper security mechanisms, an attacker could manipulate or forge data, leading to misleading measurements and unreliable analyses. This not only affects the accuracy of the collected information but also undermines trust in the entire system. Despite the clear risks, security considerations remain largely overlooked in the current state of the art. We point out the most severe in the following and we picture the described attack surface in Figure 2.

#### 3.2.1. Exploitation of Firmware Vulnerabilities

The firmware of an IoT device may present security vulnerabilities that can be exploited to tamper with its functionalities and compromise the device. Specifically, the firmware update procedure can become an attacker’s critical point of entry, a problem whose severity increases without client-side validation of the received firmware. A common strategy is using OTA (Over-The-Air) updates, which feature packed files containing the updated information of the modified firmware components. Attackers could inject malicious artefacts into the device’s code if this process is not correctly validated. Additionally, authentication is another critical factor: developers often implement security via a traditional *user:password* tuple that is often hardcoded into the device. Attackers are often able to dump and reverse-engineer the firmware to extract such hardcoded credentials, leading to potential data breaches.

#### 3.2.2. Malware Attacks

Unprotected IoT devices are highly susceptible to malware attacks. Ref. [41] provides a detailed illustration of the attack chain of *Mirai*, the most prominent malware family used to target and infect IoT devices. Mirai operates as a botnet to perform, among other things, coordinated DDoS attacks on specific targets. It employs a brute-force approach to scan for exposed vulnerable services and recruits compromised devices as bots within its network.

#### 3.2.3. Insecure Communication

Message Queuing Telemetry Transport (MQTT) is one of the most widely used protocols for inter-device IoT communications. This protocol is popular due to its lightweight nature and low latency. However, in its default version, MQTT lacks built-in security measures. Ref. [42] provides a comprehensive survey of the current security challenges in MQTT communications. For example, the protocol is susceptible to Man-in-the-Middle (MITM) attacks because MQTT communications are not encrypted by default. Therefore, attackers may intercept the transmitted message and steal or corrupt its content. Another commonly used communication methodology is based on RESTful APIs. If unprotected, these APIs can be exploited to inject or leak data. Implementing robust access-control mechanisms for RESTful APIs is challenging in the IoT landscape and needs to be addressed in current state-of-the-art solutions.

#### 3.2.4. Data Poisoning

Machine learning approaches are increasingly applied in IoT systems. However, using received data to train models introduces vulnerabilities to data poisoning attacks, illustrated by [43]. These attacks feed malformed data to a machine learning system, compromising its performance, leading to the need of validation mechanisms to mitigate the impact of these attacks.

#### 3.2.5. Data Security

Finally, the data on which MCS applications rely is susceptible to de-anonymization, theft, and misuse. For example, an attacker may divulge data without the owner’s consent with defamatory intent. This scenario is aggravated if the said attacker can craft pieces of data that are false in content but valid in format to taint the reliability of the aggregated results. The data privacy problem has been studied, among others, by Pinto et al. [44], who analyzed multiple works proposing mitigations for privacy violations. In particular, the influence of the Solid platform (https://github.com/solid, accessed on 2 June 2025) has been noteworthy. In this respect, Ghayvat et al. [45] employed the Solid pods to store user information securely.

## 4. Towards Distributed Air-Monitoring Systems: Open Challenges and Opportunities

Despite the previously described approaches, air pollution monitoring using portable or distributed networks of sensors still presents several open challenges. Specifically, the contributions described in Section 3.1 exhibit several limitations and areas for improvement. One of the most significant issues is the lack of user incentives to participate in these crowdsensing systems. Without motivational structures, these platforms often fall into disuse shortly after their launch. Additionally, few studies address the security of the data collection process, leaving systems vulnerable to false data injection and inaccurate information. Furthermore, another challenge lies in the energy consumption of the devices used in crowdsensing. Many of these studies do not adequately consider the battery drain that participation in such systems can impose on user devices, particularly smartphones. Since crowdsensing applications often run continuously or in the background, the increased power consumption could discourage users from participating.

In general, the effective coverage of remote areas with a significant number of sensors represents a non-trivial task. In this sense, the MCS paradigm represents a perfect solution for extending monitoring, thanks to the use of mobile devices. However, as highlighted in the previous sections, one of the major challenges is represented by the incentive for users to participate in all the illustrated crowdsensing platforms. Without engagement strategies, these applications risk being abandoned soon after deployment, limiting their effectiveness.

From this point of view, the involvement of local communities in air pollution monitoring by establishing citizen science networks could represent a more interesting approach. Citizen science networks involve volunteers in the collection and analysis of data on air quality using low-cost sensors and other monitoring equipment [46], thus representing a way to raise awareness on environmental preservation and mitigate the problem of access to specific areas. Different training levels of volunteers can be established, according to the easiness-of-use of the proposed platforms. The goal of the research is to progressively move towards platforms which require minimum interaction with the user, who has the main role of reaching the measurement area and providing the ideal condition for data acquisition.

Nonetheless, this approach strongly relies on citizens’ adherence to air pollution monitoring programs, thus ensuring the availability of a significant amount of data analysis. Indeed, portable sensors are generally characterized by lower performances in terms of sensitivity and reproducibility that can only be mitigated by significant averaging between different, independent acquisitions occurring in a given area in a limited time. Finding strategies to ensure the engagement of distributed citizen science networks is an open challenge for the actual application of distributed air-monitoring programs in real scenarios.

A possible approach in this sense is represented by gamification, which is an emerging paradigm in several fields. According to a survey on the matter redacted by [47], gamification is commonly defined as “the use of game elements and mechanics in non-game contexts”.

**Figure 3:**
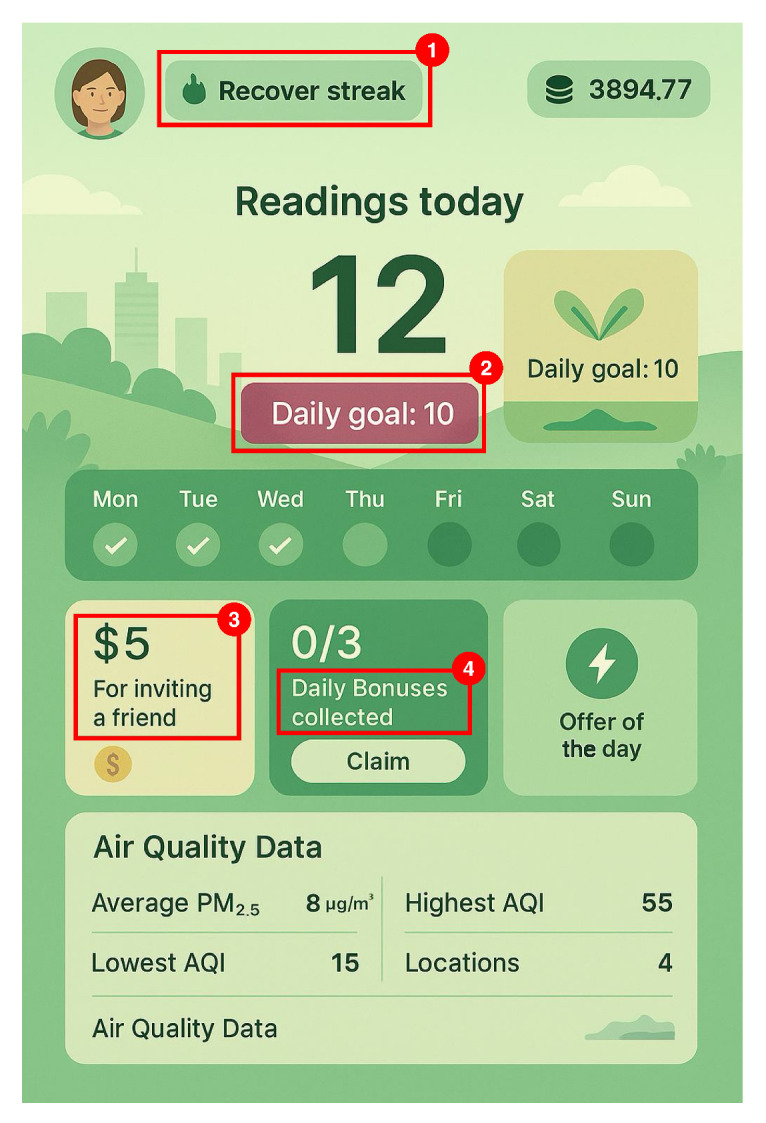
Mockup of an application to collect air quality data. It uses multiple gamification approaches, like streak loss (1), daily tasks (2), friend involvement (3), and rewards (4).

The concept of gamification is rooted in strong psychological foundations, as highlighted in [48]. Both theoretical and empirical studies have demonstrated that incorporating gamification dynamics can enhance user motivation and engagement. These dynamics align closely with Self-Determination Theory (SDT) principles, emphasizing three key psychological needs: autonomy, competence, and relatedness. By enabling users to make choices and find solutions, gamification promotes intrinsic motivation. The latter is derived from the user’s enjoyment and satisfaction in completing tasks for their inherent value rather than for external rewards. Furthermore, gamification leverages key psychological concepts such as positive reinforcement and punishment, e.g., with point systems used to encourage positive behaviors and discourage undesired ones. Finally, gamification can offer users a personalized experience, such as selecting avatars or quests, enhancing the sense of ownership and autonomy, which in turn boosts the user’s engagement. To visually depict these concepts, we present in Figure 3 a mockup of an application that offers rewards to users who collect air quality data. In the literature, we find a work that implements gamification elements in crowdsensing systems for air quality collection, specifically the AirMatter project, using a bicycle-based approach [49]. They developed a web application integrating game-like features—such as levels, experience points, and achievements—to encourage user participation and data gathering.

Although a significant gap exists between the psychological theories underpinning gamification and its actual empirical validation [47], the technique has been employed successfully numerous times.

Integrating game-like mechanics into distributed monitoring systems enhances user engagement by promoting a sense of autonomy and competence [50]. Features such as leaderboards, rewards, and challenges encourage participation by making the process engaging and rewarding. For instance, using mobile apps that reward users for measuring air quality in their neighbourhoods increases participation and user involvement [51]. This factor increases the user’s willingness to engage with the system, which is a critical factor in applications that require continuous monitoring. For example, Ref. [52] used gamification to monitor health-related routines in 520 adults during a time span of three months. In the case of mobile objects equipped with air quality sensors, gamification could encourage users to travel specific routes, earning points for contributing to data collection in under-monitored and more critical areas. Examples of successful applications include recycling initiatives where users earn rewards for proper waste disposal [53]. Similarly, in the context of air quality, volunteers could receive badges for reaching milestones, such as recording data from a certain number of unique locations, or for long-term participation in monitoring activities.

Furthermore, an essential aspect of gamification is its ability to promote a sense of community and collective action. Research highlights that promoting social interactions, such as sharing achievements or engaging in friendly competitions, can substantially boost engagement [54]. For instance, organizing challenges to identify the “most active participant” motivates participation. Moreover, trust in the data and the system can be improved through transparent gamification elements [55]. For instance, leaderboards that display real-time contributions and visualize data trends can demonstrate the collective impact of monitoring efforts. Additionally, participants can be educated about the reliability and calibration of sensors through gamified tutorials or challenges that explain technical details in an accessible way. The resulting collective action may also be used to better existing official databases, as seen in [56] with the case of FotoQuest Go. This application was aimed at in situ data collection: volunteers would collect data about land use and land cover (LULC) in the field using a mobile device. Data collected through this approach was used to improve the official LUCAS (https://ec.europa.eu/eurostat/statistics-explained/index.php?title=Glossary:Land_use/cover_area_frame_survey_(LUCAS), accessed on 2 June 2025) data.

## 5. Conclusions

In this paper, the most recent approaches to the employment of crowdsensing systems in the monitoring of air pollution have been discussed. The problem of the main constituents of outdoor air pollution has been presented, and their impact on health and socio-economical fabrics has been discussed. Mobile crowdsensing (MCS) has emerged as a promising paradigm, allowing citizens, vehicles, and IoT devices to contribute to real-time air quality measurements with high spatial resolution, significantly reducing costs compared to traditional monitoring stations. In this sense, crowdsensing can be seen as a complementary approach to standard monitoring networks, better suited for extending the spatial and temporal coverage of air quality evaluation towards personalized exposure assessment, while fixed stations can be used for regulatory reasons thanks to their high reliability in the long term. Moreover, cost-effectiveness can ensure the establishment of monitoring frameworks in regions, such as developing countries, in which fixed station installation and maintenance could not even be afforded. Despite its potential, the widespread adoption of crowdsensing faces critical challenges. The first aspect is related to data robustness and reliability, which suffer from the ineherent limited sensitivity and reliability of low-cost, miniaturized pollution sensors currently available on the market. Although higher spatial and time resolution can be effectively employed to mitigate this potential issue by averaging approaches, the integration of crowdsensed data into national and global monitoring frameworks will require significant effort in terms of testing, calibration, and validation. Similarly, ensuring data security and reliability represents an open issue. Security vulnerabilities in firmware, communication protocols, and data integrity expose these systems to risks like data poisoning, malware attacks, and unauthorized manipulation. Addressing these barriers requires implementing robust authentication, encrypted data transmission, and anomaly detection algorithms to ensure the trustworthiness of the collected data. This field of research is relatively recent and has strong potentialities that evolve with IoT technologies and devices; for instance, increasing public engagement through gamification strategies—such as leaderboards, rewards, and community challenges—can enhance participation and improve the effectiveness of distributed air quality monitoring systems. To date, such potentialities are barely explored, thus representing a possible future trend for research and industry with high benefits for society and public health.

## Figures and Tables

**Table 1:** Air quality thresholds from EU and WHO.

Pollutant	EU Limit	WHO Guideline (2021)	Averaging Time
PM_2.5_	25 µg/m^3^	5 µg/m^3^	Annual
PM_10_	40 µg/m^3^	15 µg/m^3^	Annual
NO_2_	40 µg/m^3^	10 µg/m^3^	Annual
O_3_	180 µg/m^3^	100 µg/m^3^	8-h mean
SO_2_	125 µg/m^3^	40 µg/m^3^	24-h mean
CO	10 mg/m^3^	4 mg/m^3^	8-h mean

**Table 2:** Analysis of the most recent MCS approaches in the state of the art.

Ref.	Objects	Collected Data	Used Devices	Data Utilization	User Involvement
[30]	Vehicles (buses, cars)	PM_10_, PM_2.5_, geolocation	Nova SDS011 (Nova Fitness Co., Jinan, China)	Web-based visualization	Participatory
[31]	Vehicles (buses)	PM_10_, PM_2.5_	Nova SDS011 (Nova Fitness Co., Jinan, China)	Web-based visualization, statistical correction	Participatory
[32]	Personal vehicles and traffic sensors	NO_2_, CO, PM_x_, noise	Custom board, sensors not specified	Web-based visualization, prediction	Opportunistic
[33]	IoT devices	NO_2_, PM, personal exposure	Alphasense CO-A4, NO2-A43F and OX-A431 (Alphasense Ltd., Essex, UK)	Web-based visualization, prediction	Participatory
[34]	Shared bikes, IoT devices	PM_2.5_, CO, NO_2_	Custom box based on Arduino, sensors not specified	Missing data estimation, web-based visualization	Opportunistic
[35]	IoT devices	PM, air pollution	Custom box with ESP8266 microcontroller (Espressif Systems, Shanghai, China), PMS 5003 sensor (Plantower, Beijing, China), and SHT31-D sensor (Sensirion AG, Stäfa, Switzerland)	Web-based visualization, data stories	Participatory
[36]	Smartphones	Noise levels	Smartphone built-in microphone	Web-based analysis and support system	Participatory
[37]	Smartphones (battery sensors)	Temperature	Smartphone battery temperature sensor	HVAC optimization	Opportunistic
[38]	IoT devices	CO_2_, NO_2_, PM_10_, PM_2.5_	MiCS5524 (SGX Sensortech, Neuchâtel, Switzerland), Plantower PM2.5 (Plantower, Beijing, China), and MH-Z19B (Winsen Electronics, Zhengzhou, China)	Prediction, early warnings	Opportunistic
[39]	IoT devices	Temperature, humidity, CO_2_	Sensors not specified	Missing data estimation, web-based visualization	Opportunistic
